# Lipoprotein biology during induced oogenesis in the short-finned eel, *Anguilla australis*—hepatic target gene expression and accumulation of nutrient sources into developing oocytes

**DOI:** 10.1007/s10695-026-01806-w

**Published:** 2026-10-03

**Authors:** Erin L. Damsteegt, Benedetta Mercuriali, Georgia Thomson-Laing, Marin Miner, Anh Tuan Nguyen, P. Mark Lokman

**Affiliations:** 1 Department of Zoology, University of Otago, 340 Great King Street, Dunedin, 9016 Aotearoa New Zealand; 2 Department of Pharmaceutical Sciences, University of Milan, Milan, 20133 Italy; 3 Coastal and Freshwater Group, Cawthron Institute, 98 Halifax Street, Nelson, 7010 Aotearoa New Zealand; 4 Oregon Health and Science University, 3181 SW Sam Jackson Park Road, Portland, Oregon, 97239 USA; 5 Department of Aquaculture, University of Agriculture and Forestry, Hue University, 6 Le Loi Street, Hue, 530000 Vietnam

**Keywords:** Artificial maturation, Hypophysation, Lipoproteins, Liver, Ovary, Lipid packaging, Anguilla

## Abstract

Production of good quality eggs still plagues mass propagation efforts of anguillid eels. Understanding the lipid dynamics of artificially maturing eels will inevitably benefit captive rearing practices due to the importance of lipids for egg quality. Fortnightly sampling of shortfinned eels (*Anguilla australis*) subjected to hypophysation for 10 weeks allowed us to gather important information relating to multiple aspects of lipid physiology. Accordingly, expected increases in gonadosomatic (from 2.20 ± 0.14 to 23.20 ± 6.01%) and hepatic somatic indices (from 0.89 ± 0.04 to 1.93 ± 0.01%), as well as oocyte diameter (from ~ 220 to ~ 700 µm) were complemented with notable increases in hepatic and ovarian vitellogenin levels at both the gene and protein levels in treated eels compared to Ringer-injected controls. A fivefold increase in hepatic expression of oestrogen receptor-alpha, but not the -beta isoform, corroborates the role for estradiol in promoting vitellogenesis. Conversely, decreases in expression of apolipoprotein b and the peroxisome proliferator-activated receptors in the liver point to a change in the role of the liver away from the neutral lipid packaging associated with previtellogenesis towards vitellogenin loading, ultimately culminating in yolk protein sequestration by the oocytes.

**Supplementary information:**

The online version contains supplementary material available at https://doi.org/10.1007/s10695-026-01806-w.

## Introduction

The elusive nature of anguillid eels following the commencement of their oceanic migration makes researching the later stages of oogenesis inherently difficult. When most temperate anguillid species leave their freshwater growth habitats, their oocytes are only just starting to sequester vitellogenin. Furthermore, the gonadosomatic indices of these eels range between 3 and 10% (see Boetius et al. 1962 [*Anguilla anguilla* and *A. rostrata*]; Yamamoto and Yamauchi 1974 [*A. japonica*]; Dorairaj and Nandakumar 1982 [*A. bicolor*]; Todd 1974; Lokman et al. 1998 [*A. australis* and *A. dieffenbachii*]), a long way short of the 35–50% of the individual’s body weight that would usually be accounted for by the fully mature ovary (as estimated from induced spawning trials). Even in tropical anguillid eels which are often considered to be developmentally advanced when they leave the continental waters (potentially due to their closer proximity to spawning grounds), oocytes are only in the early vitellogenic stage (*Anguilla bicolor pacifica*, Hagihara et al. 2012; 2020). To study the later stages of oogenesis, researchers are left with two options: either track, capture and sample eels along their migration routes or induce ovarian maturation in captivity. Inevitability the second option is favoured, not in the least because of the decades of efforts already put into optimising artificial maturation protocols (see Yamamoto and Yamauchi 1974; Lokman and Young 2000; Palstra et al. 2005; Dou et al. 2008; Oliveira and Hable 2010; Burgerhout et al. 2011; Mordenti et al. 2013; Lokman et al. 2015; Mes et al. 2016; Di Biase et al. 2017; Damsteegt et al. 2020).

Faced with the comprehensive decline in wild eel stocks, the reliable and cost-effective propagation of captive eels is becoming ever more urgent. In the last decade, several key difficulties impeding mass production of freshwater eels have been highlighted: obtaining quality broodstock, producing good quality eggs (Heinsbroek et al. 2013), optimising larval/leptocephali food and environments (Benini et al. 2023; Hatakeyama et al. 2023), simplifying and shortening the larval/leptocephali rearing period, disease prevention and reducing morphological abnormalities (Sørensen et al. 2014; Jéhannet et al. 2024). Arguably the most important bottleneck to overcome is the production of good quality eggs due to the flow-on effects of improved hatch rates and fitter, healthier larvae with fewer morphological irregularities. The significance of lipids in terms of egg quality for marine spawning fish has been emphasised numerous times (reviewed in Rainuzzo et al. 1997; Izquierdo et al. 2001; Reading et al. 2018); yet, other than a single publication (Ozaki et al. 2008), almost nothing is known about any aspect of lipid physiology in eel broodstock used for propagation. Basic information on lipid dynamics during artificial maturation is therefore imperative.

Following on from our previous study (Damsteegt et al. 2025) in which we examined several key aspects of blood biochemistry (i.e. sex steroid concentrations, lipoprotein profiles and cholesterol and triacylglyceride levels) throughout artificial maturation, we investigated the molecular mechanisms underlying lipid transport and utilisation during oogenesis. In teleosts, lipids destined for developing oocytes are synthesised and packaged in the liver before being transported in the circulation and subsequently being taken up by the ovary (reviewed in Tocher 2003). Members of the large lipid transfer protein (LLTP) family, including apolipoprotein B (Apob), microsomal triglyceride transfer protein (Mttp) and vitellogenin (Vtg), play central roles in this process, with Apob and Mttp involved in hepatic lipoprotein assembly and Vtg mediating the transfer of lipid and other nutrients to developing oocytes. Apolipoprotein E (Apoe), a protein with an array of functions in mammals (Marais 2019), also contributes to lipoprotein transport and uptake into target tissues. The peroxisome proliferator-activated receptors (Ppars) are ligand-activated transcription factors that act as important regulators of hepatic lipid metabolism by, in mammals, stimulating fatty acid use (PPARα) or storage (PPARg; Berthier et al. 2021), and may therefore influence lipid availability for ovarian development. Furthermore, oestrogen and androgen, signalling through Esr1, Esr2, Ara and Arb, regulate aspects of both hepatic and reproductive function.

Accordingly, we quantified mRNA and/or protein levels of *apob*, *mttp* and *vtg* in the liver and/or ovary, and assessed hepatic expression of *apoe*, *ara*, *arb*, *esr1*, *esr2* and members of the Ppar family (*ppara*, *pparb* and *pparg*). Given the essential roles of LLTP proteins in lipoprotein assembly, transport and delivery to target tissues (e.g. Smolenaars et al. 2007), we also conducted promoter analysis to gain insight into the potential regulation of these genes during artificial maturation.

## Materials and methods

### Animals and experimental design

Animal husbandry and induction of oogenesis via salmon pituitary homogenate (SPH) injection are described in Damsteegt et al. (2025). In brief, 25 early pubertal female shortfinned eels (*Anguilla australis*) were subjected to weekly injections with 10 mg/kg SPH to induce oogenesis. A further five eels received weekly injections of Ringer (saline) solution and served as control animals.

### Terminal sampling

Five fish were euthanised in benzocaine (0.3 g/l) at time-zero and used as initial controls, a further five fish treated with SPH were euthanised every two weeks for 10 weeks. At 10 weeks, an additional five fish that had been treated with weekly Ringer injections were euthanised. Following tail removal, the blood from the caudal vein was collected, mixed with ca. 1% (v/v) ethylenediaminetetraacetic acid (200 mg/mL) and centrifuged (10,000* g* for 5 min). The isolated plasma was used to measure concentrations of 11-ketotestosterone (11-KT) and estradiol-17β (E_2_) by radioimmunoassay and to obtain fast-protein liquid chromatography profiles, triacylglyceride and cholesterol measurements and conduct vitellogenin and apolipoprotein B protein quantification; these results were presented and discussed in Damsteegt et al. (2025). The liver, left eye and right ovary were removed and weighed for calculation of somatic indices (organ weight as a percentage of body weight). Portions of liver and ovary were either fixed in Bouin’s solution for histological examination (Histology) or flash frozen on dry ice for quantification of vitellogenin and apolipoprotein B via slotblot (Slotblot assay for apolipoprotein B and vitellogenin levels in liver and ovary) and of target gene expression in the liver (Candidate gene expression in liver during induced oogenesis). Another portion of ovary was submerged in eel Ringer containing collagenase (1 mg/ml) for oocyte diameter measurements (oocyte diameter data have been published in Babio et al. 2023 and Damsteegt et al. 2025).

### Histology

Bouin’s-fixed liver and ovary samples were embedded in paraffin, sectioned (5 µm) and stained with haematoxylin and eosin. Micrographs were taken with a compound microscope mounted with an Olympus SC100 camera. Liver micrographs were subjected to white space analysis in ImageJ (version: 1.53 g). Prior to analysis, all micrographs were converted to 8-bit greyscale, contrast was enhanced and histogram normalisation was performed. The hepatic white space (HWS) was then measured across four different sections of the micrograph, ensuring only hepatocytes were included, before the average HWS, as a percentage of total surface area, was calculated. HWS was used as a proxy for intra-hepatocyte lipid content.

### Slotblot assay for apolipoprotein B and vitellogenin levels in liver and ovary

Frozen liver and ovarian tissue samples were homogenised by hand in 1 × cOmplete ULTRA Mini Protease inhibitor (Roche) in phosphate-buffered saline, then centrifuged at 4 °C and 10,000* g* for 60 min. Vtg and Apob content in the supernatant were measured via immunoreactive slotblot as described in Damsteegt et al. (2025).

### Candidate gene expression in the liver during induced oogenesis

#### RNA extraction, DNase treatment, RNA quantification and cDNA synthesis

Total RNA was extracted from 50 to 100 mg of hepatic tissue using TRIzol reagent (Invitrogen) following the manufacturer’s instructions. After RNA quantification by spectrophotometry, 5 μg of total RNA was treated with TURBO DNA-free (Ambion), to reduce genomic DNA contamination. The concentration of DNase-treated RNA was determined using a Quant-it RiboGreen RNA assay kit (Invitrogen), after which complementary DNA (cDNA) was synthesised by reverse transcribing 500 ng of total RNA (PrimeScript RT reagent Kit; Takara) according to the manufacturer’s protocol.

#### Quantification of candidate gene transcript abundance by qPCR

Standards for each qPCR target gene were made from serial dilutions of PCR product previously extracted from agarose gel (~ 500 pb) and confirmation sequenced exactly as described in Damsteegt et al. (2014) (see supplementary Table 1 for standard PCR primer details). All qPCR reactions were performed on a QuantStudio 5 thermal cycler (Applied Biosystems) and carried out in keeping with the guidelines for the minimum information for publication of quantitative real-time PCR experiments (MIQE: Bustin et al. 2009). Each 10-μL qPCR reaction consisted of 2 μL cDNA (10 ng/μL), 0.5 μL of each primer (10 mM; see supplementary Table 2 for qPCR primer details), 5 μL TB Green™ Premix Ex Taq™ II (Tli RNase H Plus) (Takara) and 2 μL milli-Q water. Samples were run alongside four no-template controls and a 5–7-point standard curve, in duplicate. Assay specificity was confirmed via melt curve analysis and efficiency of amplification ranged from 96.1 to 101.5% (see supplementary Table 2). Target gene expression was normalised over the geometric mean of *ef1a* and *rpl36* as this combination of genes showed stable expression throughout the experiment (data not shown).

### *In silico *promoter region analysis

Putative promoter regions for the LLTPs, i.e. *apob*, *mttp*, *vtgAa* and *vtgAb*, were obtained from the genome assemblies of the European and Japanese eel (NCBI accession numbers: JABVMG000000000 and JANEZO010000000, respectively). For each gene in each species, a 2-kb region upstream of the annotated start codon was retrieved for analysis.

Canonical oestrogen response elements (EREs) were defined according to the palindromic consensus sequence 5’-GGTCAnnnTGACC-3’, and canonical androgen response elements (AREs) as 5’-GGTACAnnnTGTTCT-3’. To account for natural sequence variation, both perfect palindromes and degenerate motifs with up to two mismatches were considered. Only perfectly matching half-sites were deemed acceptable.

Position weight matrices (PWMs) for human oestrogen receptor-1 and −2 and the androgen receptor were retrieved from the JASPAR 2024 database (matrix IDs MA0112.2, MA0258.2 and MA0007.2, respectively). PWM-based motif scanning was performed using FIMO (Find Individual Motif Occurrences; MEME Suite v5.5.8, Grant et al. 2011), with a significance threshold of *p* < 0.001. The FIMO background model was based on the input sequence composition. Candidate sites were ranked according to PWM score and associated *p*-values, and significance was determined following false discovery rate (FDR) correction using the Benjamini–Hochberg procedure (*q* < 0.05).

### Statistical analysis

To account for inter-individual variation in responsiveness to SPH treatment, data were plotted against oocyte diameter rather than time post-injection. Data normality and homogeneity of variance was checked using Shapiro–Wilk and Levene’s tests respectively. Correlations between oocyte diameter (log-transformed) and HSI, HWS, eye index, protein levels or gene expression data were subjected to regression analysis. Differences between the initial sampling point (0 weeks) and the termination of the experiment (10-week Ringer) were identified using independent *t*-tests with a Bonferroni adjusted *p* value. Statistical tests were conducted in GraphPad Prism version 10.1.0.

## Results

A liver sample from the 6-week terminal sampling was lost during RNA processing. Sample size in the 8- and 10-week SPH-treated groups was reduced from five to four after contaminated seawater was inadvertently added to the stock tank (see Damsteegt et al. 2025). Means and standard errors of oocyte diameter for each treatment group and the correlation between GSI and oocyte diameter have been reported previously (Damsteegt et al. 2025).

### Morphometrics and histology

**Fig. 1:**
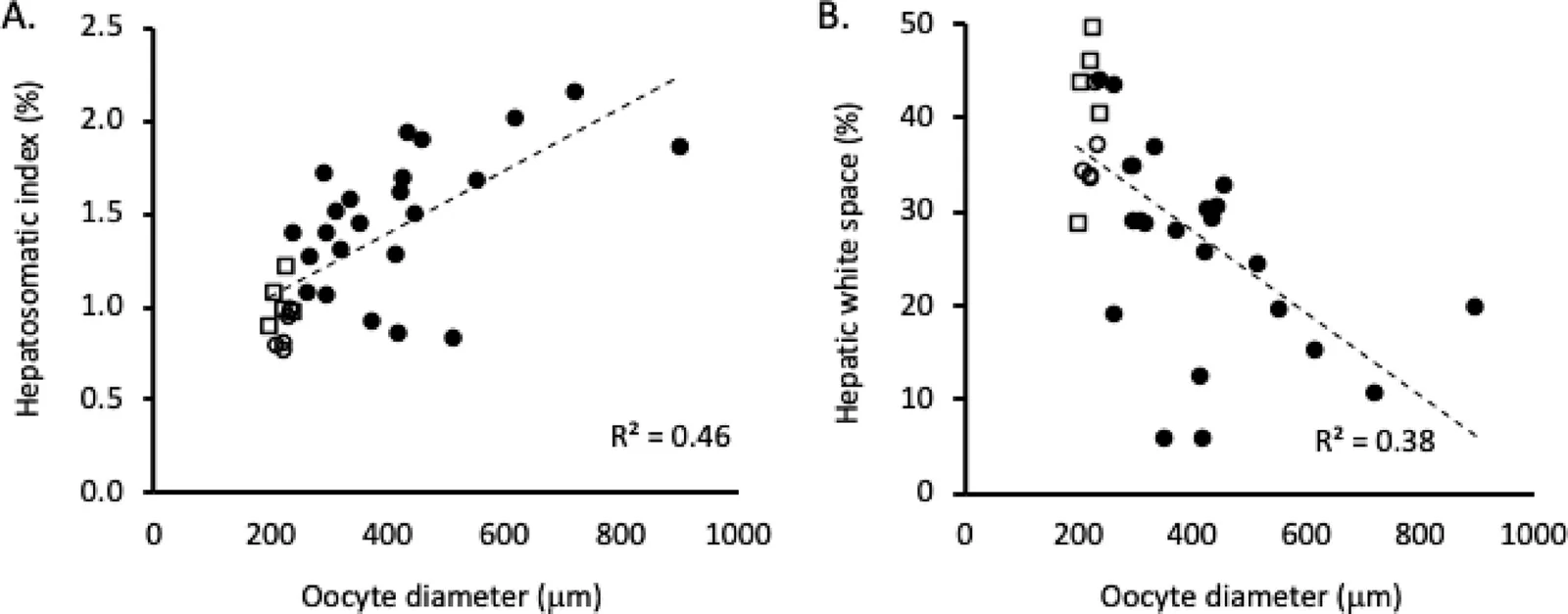
Relationship between oocyte diameter and hepatosomatic index (**A**) and hepatic white space (**B**) in female short-finned eels (*Anguilla australis*). Eels were treated for 10 weeks with either 10 mg/kg salmon pituitary homogenate (filled circles) or Ringer (open squares); initial controls are indicated by open circles. Sample size: *n* = 4 (8, 10 weeks) or *n* = 5 (all other sampling times). Trendlines are present only when significant correlations were detected (*p* < 0.05)

No difference in eye index was detected as oocyte diameters increased or when comparing between initial time-zero fish and fish treated with Ringer for 10 weeks (Supplementary Fig. 1 A). In contrast, oocyte diameter and HSI were highly correlated (Fig. 1; *p* < 0.0001). Whilst HSIs of initial control and Ringer-treated fish averaged 0.95 ± 0.04%, individuals treated with SPH for 10 week had HSIs more than twice this value. Meanwhile, HWS decreased (*p* < 0.0001) from approximately 40% in fish with oocytes around 200 μm to less than 20% in fish with oocytes greater than 550 μm.

### Liver and ovary histology

**Fig. 2:**
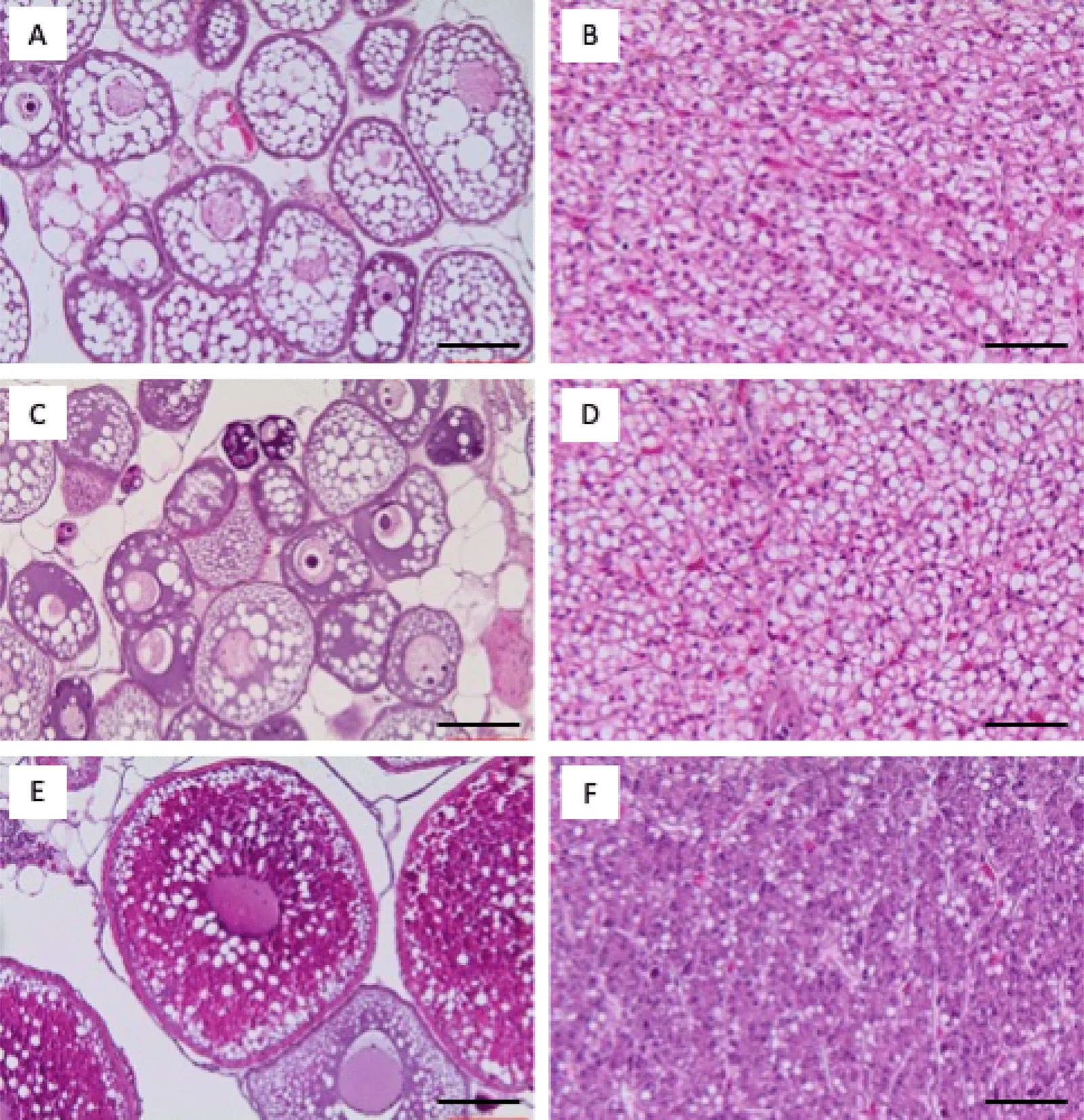
Micrographs of ovarian and hepatic tissue from female short-finned eel, *Anguilla australis*. Each micrograph pair is taken from the same fish and is representative of the time-zero control fish (**A** and **B**), fish treated for 10-weeks with Ringer’s (**C** and **D**) or fish treated for 10-weeks with 10 mg/kg salmon pituitary homogenate (**E** and **F**). Scale bars: 100 µm on ovarian micrographs and 50 µm on micrographs of liver

Accumulation of yolk was not evident in the oocytes of the time-zero control fish (Fig. 2A) nor in the fish treated with Ringer for 10 weeks (Fig. 2C). After 4 weeks of SPH treatment, when oocytes were reaching 300 μm in diameter, yolk granules could be seen around the periphery of the oocytes from four of the five fish sampled. Yolk was rapidly accumulated within the oocytes throughout the remainder of SPH treatment with tightly packed yolk granules dominating the ooplasm after 10 weeks (Fig. 2E). The liver from both the time-zero control fish (Fig. 2B) and the fish treated with Ringer for 10 weeks (Fig. 2D) was dominated by lipid, most of which had disappeared by 10 weeks of SPH treatment (Fig. 2F).

### Hepatic and ovarian estimates of apolipoprotein b and vitellogenin

**Fig. 3:**
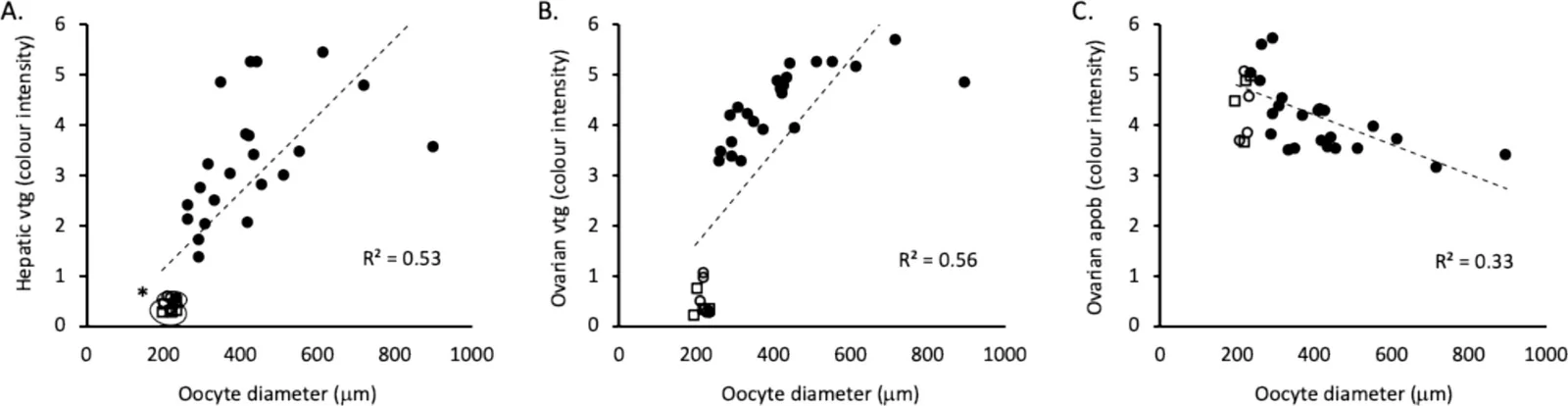
Relationship between oocyte diameter and hepatic (**A**) and ovarian (**B**) vitellogenin protein and ovarian apolipoprotein b protein (**C**) in female short-finned eels (*Anguilla australis*). Eels were treated for 10 weeks with either 10 mg/kg salmon pituitary homogenate (filled circles) or Ringer (open squares), initial controls are indicated by open circles. Sample size: *n* = 4 (6, 8 and 10 weeks) or *n* = 5 (all other sampling times). Trendlines are present only when significant correlations were detected (*p* < 0.05). * indicates a significant difference between Ringer-treated eels and initial controls (*p* < 0.05) with respective groups enclosed by circles

Vitellogenin levels increased significantly in both the liver (*p* < 0.001) and ovary (*p* < 0.001) as oocyte diameter increased (Fig. 3A and B respectively). Concentrations of vitellogenin in the liver of initial control fish averaged 0.5 arbitrary units (AU) and increased more than eightfold in fish with the largest oocytes. A similar, but sharper, increase was seen in ovarian vitellogenin levels. Indeed, hepatic and ovarian vitellogenin concentrations were highly correlated (*p* < 0.0001, *R*^2^ = 0.77, data not shown). No differences were found in hepatic Apob levels (Supplementary Fig. 1B), but a steady decrease was detected in ovarian Apob levels (*p* < 0.001) (Fig. 3C) as oocyte diameters increased.

### Candidate gene expression in eel liver

#### LLTP family members (apob, mttp, vtgAa and vtgAb) and apoe

**Fig. 4:**
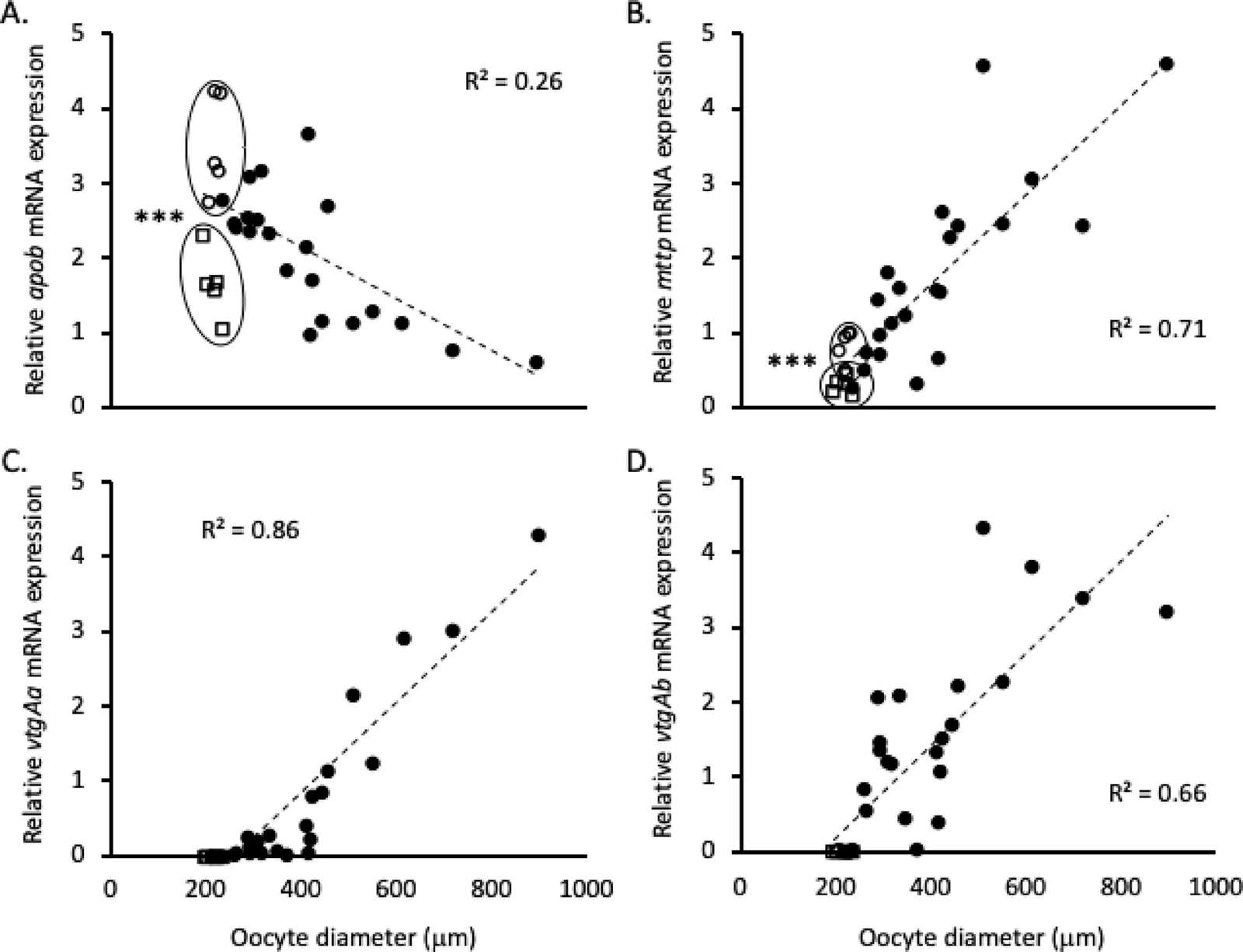
Relationship between oocyte diameter and relative mRNA expression of apolipoprotein b (**A**), microsomal triglyceride transfer protein (**B**), vitellogenin Aa (**C**) and vitellogenin Ab (**D**) in the liver of female short-finned eels (*Anguilla australis*). Eels were treated for 10 weeks with either 10 mg/kg salmon pituitary homogenate (filled circles) or Ringer (open squares), initial controls are indicated by open circles. Sample size: *n* = 4 (6, 8 and 10 weeks) or *n* = 5 (all other sampling times). Trendlines are present only when significant correlations were detected (*p* < 0.05). *** indicates a significant difference between Ringer-treated eels and initial controls (*p* < 0.001) with respective groups enclosed by circles

A significant decrease (*p* < 0.01) in hepatic *apob* mRNA expression was evident with increased oocyte diameter (Fig. 4A). Levels of *apob* mRNA were almost fourfold lower in livers of fish treated with SPH for 10 weeks and with oocytes more than 550 µm, compared to levels in the time-zero control fish with oocytes around 200 µm. Fish treated with Ringer for 10 weeks had levels of *apob* mRNA approximately half those of the time-zero fish (*t* = 5.243, *p* < 0.001). No changes in *apoe* expression were detected (Supplementary Fig. 1 C).

Hepatic *mttp* expression significantly decreased (*t* = 5.194, *p* < 0.001) between time-zero fish and fish treated with Ringer for 10 weeks (Fig. 4B). However, *mttp* mRNA levels increased significantly with increasing oocyte diameter (*p* < 0.0001). Levels of expression were 3–fivefold higher in fish with the largest oocytes compared to fish with the smallest oocytes.

Hepatic expression of both *vtgAa* (*p* < 0.0001) and *vtgAb* (*p* < 0.0001) increased significantly with increasing oocyte diameter. The expression of both vtg genes increased after only 2 weeks of SPH treatment and continued increasing throughout the treatment period. No difference was detected in expression levels of either gene between time-zero fish and fish treated with ringer for 10 weeks.

Of note, *vtgAb* was more highly expressed than *vtgAa*, reaching critical cycle thresholds, on average, four cycles earlier. The biggest difference in Cq value was in the time-zero fish where *vtgAb* reached the critical cycle threshold an average of nine cycles earlier than *vtgAa*, this gap was reduced to less than one Cq value in the fish treated with SPH for 10 weeks. Despite the difference in expression levels, hepatic mRNA levels of *vtgAa* and *vtgAb* were strongly correlated (*p* < 0.0001, *R*^2^ = 0.69; data not shown).

#### Peroxisome proliferator-activated receptors (ppara, pparb and pparg)

**Fig. 5:**
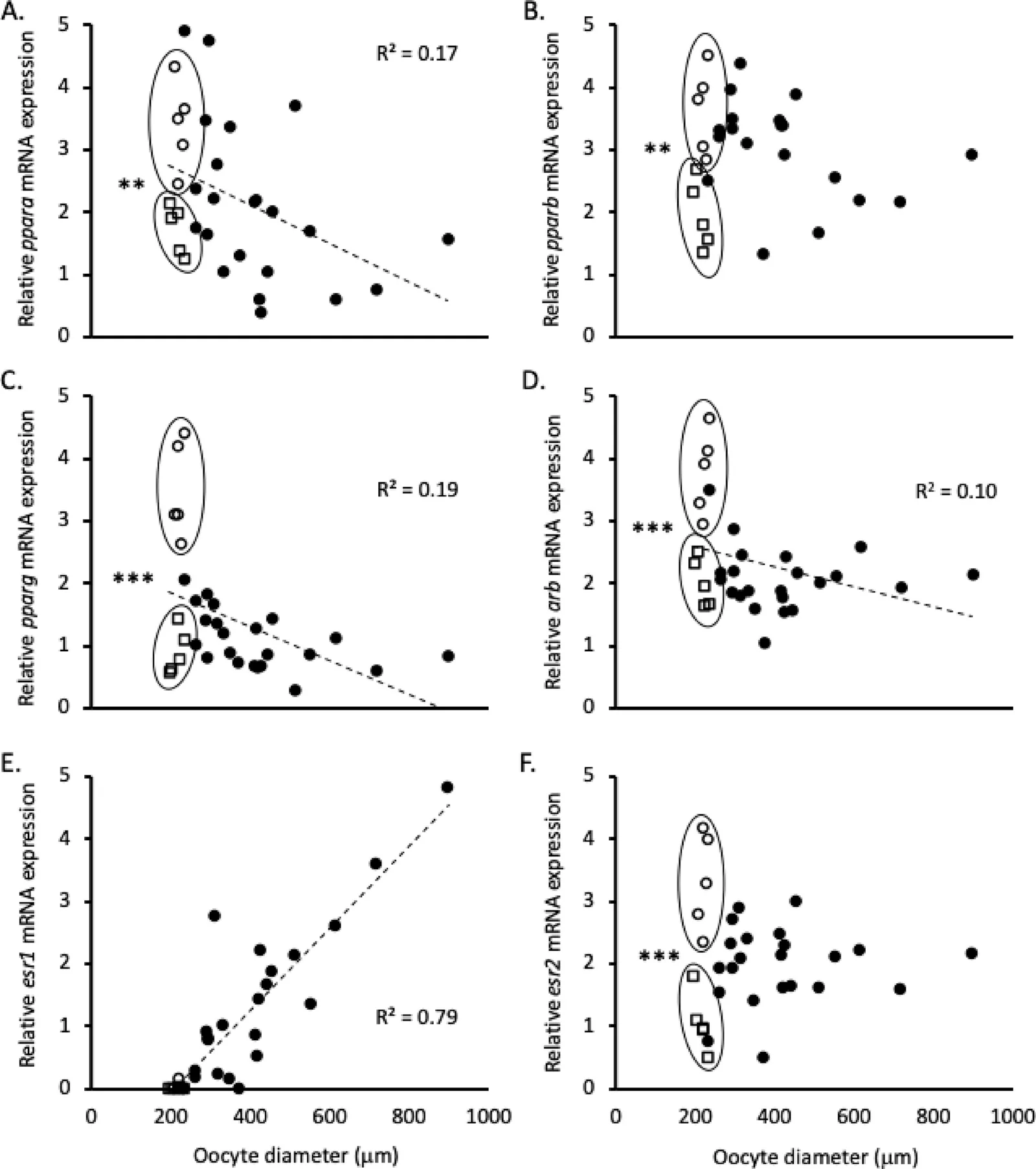
Relationship between oocyte diameter and relative mRNA expression of peroxisome proliferator-activated receptors alpha (**A**), beta (**B**) and gamma (**C**), androgen receptor beta (**D**) and the alpha (**E**) and beta (**F**) oestrogen receptors in the liver of female short-finned eels (*Anguilla australis*). Eels were treated for 10 weeks with either 10 mg/kg salmon pituitary homogenate (filled circles) or Ringer (open squares), initial controls are indicated by open circles. Sample size: *n* = 4 (6, 8 and 10 weeks) or *n* = 5 (all other sampling times). Trendlines are present only when significant correlations were detected (*p* < 0.05). **, *** indicate significant differences between Ringer-treated eels and initial controls (*p* < 0.01 and *p* < 0.001. respectively) with respective groups enclosed by circles

A significant reduction in the expression of both *ppara* (*p* < 0.05) and *pparg* (*p* < 0.05) was seen in the liver as oocyte diameter increased (Fig. 5A and C respectively). A similar reduction in mRNA levels was seen for both genes in the livers of fish treated with Ringer for 10 weeks when compared to levels in livers of time-zero control fish (*ppara*: t = 4.603, p < 0.001; *pparg*: t = 6.761, *p* < 0.001).

No difference in expression of *pparb* was detected following treatment with SPH (Fig. 5B); however, a significant reduction in expression was found between time-zero fish and fish treated with Ringer for 10 weeks (*t* = 4.296, *p* < 0.001).

#### Androgen and oestrogen receptors (ara, arb, esr1 and esr2)

Expression of both *arb* and *esr2* in the liver was significantly lower in fish treated with Ringer for 10 weeks than in initial control fish (*arb*: *t* = 5.017, *p* < 0.001, Fig. 5D; *erb*: *t* = 5.575, *p* < 0.001, Fig. 5F). A significant (*p* < 0.001) and highly correlated (*R*^*2*^ = 0.79) increase in hepatic *esr1* expression was seen with increasing oocyte diameter (Fig. 5E). No significant changes in *ara* expression were detected at any point during the time trial (supplementary Fig. 1D).

### *In silico *promoter region analysis

#### Oestrogen response elements

Motif scanning of the 2-kb upstream regions of *apob*, *mttp*, *vtgAa* and *vtgAb* in European (*Anguilla anguilla*) and Japanese eel (*A. japonica*) identified multiple putative EREs (Table 1). Both full palindromic EREs and isolated half-sites were detected across all promoters (see Supplementary Fig. 2).

**Table 1:** The number of perfect oestrogen response element (ERE) half sites that were not part of a full ERE, and full ERE sites located within the 2-kb upstream region of the start codon for *apolipoprotein b* (*apob*), *microsomal triacyglyceride transfer protein* (*mttp*) and the two *vitellogenin* isoforms (*vtgAa* and *vtgAb*) in the European (*A. anguilla*) and Japanese eel (*A. japonica*). Position weighted matrix (PMW) scores and FDR-adjusted *q*-values are shown for each potential ERE

Species	Gene	perfect ERE half sites	ERE full sites	mismatches	PWM score	q-value
*A. japonica*	*apob*	4	-	-	-	-
*A. anguilla*		3	-	-	-	-
*A. japonica*	*mttp*	7	GGTCAGCT**G**GAC**T**	2	13.154	0.032*
			GGT**G**ACCTTTGACC	1	5.347	0.940
*A. anguilla*		5	GGTCACCTTTGACC	0	7.231	0.405
*A. japonica*	*vtgAa*	4	GGTCAGGTT**AC**CC	2	15.558	0.006*
			GGTCATTATG**T**CC	1	9.212	0.125
*A. anguilla*		3	GGTCAGGTT**AC**CC	2	15.558	0.006*
			GGTCATTATG**T**CC	1	9.212	0.125
*A. japonica*	*vtgAb*	2	GGT**T**AACCT**A**ACC	2	14.371	0.011*
			GGTCATTTTGACC	0	11.871	0.028*
			GGTCAACCT**C**AC**T**	2	6.500	0.270
*A. anguilla*		2	GGT**T**AACCT**A**ACC	2	14.371	0.011*
			GGTC**G**TTTTGACC	1	8.290	0.158
			GGTCAACCT**C**AC**T**	2	8.129	0.158

* indicates significant motif predictions (*q* < 0.05)

No full EREs were predicted within the *apob* promoters of either species, although several perfect half-sites (three to four per species) were present. In contrast, one statistically significant full ERE was identified within the *mttp* promoter of *A. japonica* (sequence: GGTCAGCTGGACT, *q* = 0.032), suggesting a plausible oestrogen receptor binding site. The corresponding *mttp* promoter in *A. anguilla* contained a similar motif (GGTCACCTTTGACC) but did not reach significance (*q* = 0.405).

Vitellogenin promoters contained the highest number of high-scoring EREs. In *vtgAa*, a conserved motif (GGTCAGGTTACCC) was significant in both *A. japonica* and *A. anguilla* (*q* = 0.006 in both cases), while a second, lower-scoring motif (GGTCATTATGTCC) was detected but not significant (*q* = 0.125). The *vtgAb* promoter of *A. japonica* harboured two significant EREs (GGTTAACCTAACC, *q* = 0.011; and GGTCATTTTGACC, *q* = 0.028), both of which were also present, but not statistically significant, in the corresponding *A. anguilla* promoter.

#### Androgen response elements

Motif scanning for AREs identified few potential binding motifs within the 2-kb upstream regions of the analysed genes (see Supplementary Table 3 and Supplementary Fig. 2). No perfect half-site or full AREs with less than two mismatches were detected in the *apob* or *vtgAb* promoters of either eel species. A single full ARE motif was predicted within the *mttp* promoter of both *A. japonica* and *A. anguilla*. In each species, this site exhibited two mismatches relative to the canonical palindromic consensus and produced moderate PWM scores (11.327) but was not statistically significant after FDR correction (*q* = 0.139 and 0.140). Both species also contained a single perfect ARE half-site within the *mttp* and *vtgAa* promoters. However, no additional high-confidence motifs were detected.

## Discussion

Several well documented bottlenecks within eel aquaculture, such as egg quality and larval survival, can be linked to nutrient accumulation. Here, we focused on the liver, a multi-functional organ, that plays essential roles in reproduction as well as resource allocation during arguably the most physiologically stressful time of the eel’s life: fuelling a several thousand-kilometre-long migration, provisioning the ovary with nutrients required for reproduction, and doing so whilst abstaining from food. Our data provide valuable insights into lipid dynamics during artificial maturation of eel.

Liver size (or HSI) alone makes for an interesting measurement in eels. In teleosts, HSI generally increases as oogenesis progresses (red mullet, *Mullus barbatus barbatus* (Kokokiris et al. 2014); horse mackerel, *Trachurus trachurus* (van Damme et al. 2014); rosy barb, *Pethia conchonius* (Cek et al. 2001); rabbit fish, *Siganus guttatus* (Quoc Pham and Van Nguyen 2019); triggerfish, *Balistes capriscus* (Lang and Fitzhugh 2015)), but HSI has been shown to decrease during periods of starvation (zebrafish, *Danio rerio* (Drew et al. 2008); smelt, *Hypomesus transpacificus* (Hammock et al. 2020); carp, *Cyprinus carpio*, (Blasco et al. 1992); Nile tilapia, *Oreochromis niloticus* (Elbialy et al. 2022); Siberian sturgeon, *Acipenser baerii* (Liang et al. 2017)). Eels provide a model in which oogenesis progresses in the absence of exogenous energy intake. When comparing eels in the oil droplet versus early vitellogenic stages of ovarian development, the stage of development during which eels would naturally begin abstaining from food, no differences in HSI were reported in European (Jéhannet et al. 2019) or Japanese eel (Han et al. 2003; Okamura et al. 2007). However, the HSIs of shortfinned eels in the early vitellogenic stage of development were almost double those of oil droplet-stage eels (Damsteegt et al. 2015; Jéhannet et al. 2019). In the current study, the HSI of SPH-treated fish (late vitellogenic stage oocytes) once again doubled compared to time-zero fish (early vitellogenic stage oocytes), indicating a potential fourfold increase in HSI between eels with oocytes in the oil droplet stage and those in the late-vitellogenic stage of oogenesis. This second doubling in relative liver weight can be partially explained by the packaging and removal of lighter lipid stores and the increase in production of the heavier vitellogenin particles but also by a dramatic increase in overall liver size compared to body size. The HSI observed after 10 weeks of SPH treatment is comparable to values reported for spontaneously matured European eel (*A. anguilla*) (Palstra et al. 2020) and for Japanese eel (*A. japonica*) subjected to hypophysation (Rupia et al. 2014).

In mammals, hepatic expression of the *ppars* (mainly *ppara*) has been linked to food intake. During acute food deprivation, hepatic *ppara* expression increases, allowing for lipid oxidation and ketogenesis to fuel the body, particularly the skeletal muscle (Kersten et al. 1999; Inagaki et al. 2007; Kersten 2014; Montagner et al. 2016; reviewed in Burri et al. 2010; Berthier et al. 2021). However, during times of chronic food deprivation, the metabolic response shifts and hepatic *ppar* expression decreases (Suh et al. 2022; reviewed in Preidis and Gill 2017; Sharma et al. 2024). Little is known about *ppar* expression in fish; however, during our study, we found a universal decrease in hepatic *ppar* expression, potentially indicating that eels had moved beyond an acute food deprivation response into a physiological response more in line with chronic food deprivation. This contrasts with a similar study in the European eel in which an increase in hepatic *ppara* was reported following induced maturation (Bertolini et al. 2020); no data on *pparb* or *pparg* were reported. However, in the Bertolini study, livers were sampled from fish with GSIs between 10–20% (i.e. a snapshot only); in our study, fish were more developed with GSIs reaching 40%. The decrease in *ppar* expression indicates a reduction in the production of ketone bodies, yet the loading of lipoproteins continues.

The requirement of Mttp in the assembly of apob-containing lipoproteins has been well documented amongst vertebrates (Bakillah and Abbouyi 2003; Hussain et al. 2003; Rava and Hussain 2007). More recently, its necessity in the assembly of vitellogenin has also been established (Hussain et al. 2012; Sellers et al. 2005). Our findings corroborate this as hepatic *mttp* expression increases steadily throughout artificial maturation—a period of rapid hepatic vitellogenin production. These findings are in agreement with those reported during artificial maturation of the European eel (Bertolini et al. 2020). Furthermore, both putative androgen and oestrogen response elements were identified in the promoter region of *mttp*, highlighting its responsiveness to both endocrine controllers and its importance throughout the different stages of oogenesis.

The increasing levels of hepatic *esr1* transcripts were strongly correlated with the increasing concentrations of estradiol (see Damsteegt et al. 2025; E2 concentrations were approximately 3 ng/mL in fish with oocyte diameters around 200 μm and doubled to just over 6 ng/mL in fish with oocytes greater than 500 μm) measured in the plasma, whilst no clear trend emerged in the transcript levels of hepatic *erb*. In most teleosts, Esr1 is considered the main hepatic oestrogen receptor and primary mediator of oestrogen-induced vitellogenesis (European eel, *A. anguilla* (Lafont et al. 2016; Bertolini et al. 2020); fathead minnow, *Pimephales promelas* (Filby and Tyler 2005); orange-spotted grouper (*Epinephelus coioides*; Ye et al. 2022), zebrafish (*D. rerio*; Levi et al. 2009, Chen et al. 2018) and goldfish (*Carassius auratus*; Nelson and Habibi 2010), corroborating our findings. The role of Esr2 is less clear and the interactions between the two receptors appear complex and species-specific. For instance, in some of the abovementioned species, such as the orange-spotted grouper (Ye et al. 2022), zebrafish (Zhao et al. 2019) and goldfish (Nelson and Habibi 2010), Esr2 helps promote *esr1* expression, thereby indirectly contributing to vitellogenin synthesis, but Esr1 remains the main direct mediator of vitellogenin production. In other species (rainbow trout: *Oncorhynchus mykiss* (Leaños-Castañeda and Van Der Kraak 2007); medaka: *Oryzias latipes* (Chakraborty et al. 2011; Tohyama et al. 2017)), the Esr2 has been identified as the key hepatic oestrogen receptor.

The decrease in hepatic expression of *arb* was positively correlated with the reduction in plasma 11KT concentrations (see Damsteegt et al. 2025; 11KT concentrations decreased from an average of 35 ng/mL in fish with oocyte diameters around 200 μm to 10 ng/mL in fish with oocytes greater than 500 μm), but the unchanged expression of hepatic *ara* was not. The *ara* amplified, on average, two cycles earlier than *arb* indicating ~ several-fold higher expression. Despite not being strongly correlated with 11KT levels, the higher expression of *ara* suggests that it is the key androgen receptor in the liver of shortfinned eel. Not all teleosts have two androgen receptors, but amongst those which do, there is general consensus that Ara is the main hepatic androgen receptor in rainbow trout, *O. mykiss* (Takeo and Yamashita 1999), black rockfish, *Sebastes schlegelii* (Yan et al. 2022), western mosquitofish, *Gambusia affinis* (Ogina et al. 2004) and Japanese eel, *A. japonica* (Ikeuchi et al. 2001). These studies therefore provide additional support for our interpretation that Ara is the predominant androgen receptor in the eel liver.

The reduction in plasma 11KT concentrations and concomitant increase in E2 concentrations suggest a shift in endocrine regulation of oogenesis following the onset of vitellogenesis, with E2 becoming increasingly important as oogenesis progresses. This interpretation is supported by the identification of multiple putative EREs in the promoter regions of *mttp*, *vtgAa* and *vtgAb*, all of which were significantly upregulated during the later stages of the experiment, when E2 concentrations were highest. However, the reduction in 11KT concentrations was not accompanied by a complete loss of androgenic signalling, as 11KT concentrations remained detectable throughout oogenesis. Furthermore, although hepatic *apob* expression decreased, Apob protein levels in both the plasma (see Damsteegt et al. 2025) and liver remained stable. This suggests that reduced *apob* mRNA levels do not necessarily result in reduced Apob availability for lipid transport. Together with the significant reduction in HWS throughout vitellogenesis, these findings indicate that lipid packaging, transport and uptake continue during the advanced stages of oogenesis, and suggest that 11KT may continue to contribute to lipid transport despite the increasing prominence of E2 signalling.

In summary, this study provides new insight into expression patterns of hepatic lipid transport-related genes during artificial maturation of shortfinned eel, highlighting how the liver supports the competing demands of migration and reproduction in the absence of feeding. We show that liver size increases markedly throughout vitellogenesis, coinciding with sustained lipoprotein assembly and transport despite a downregulation of hepatic *ppar* expression, indicative of a shift away from ketogenesis. At the same time, the upregulation of *mttp* and oestrogen-responsive pathways, alongside stable Apob protein levels, underscores the continued importance of lipid packaging and mobilisation to the developing oocytes. Together, these findings demonstrate that, rather than simply depleting energy reserves, the liver remains metabolically active and strategically reallocates resources to support reproductive success. This improved understanding of hepatic function during maturation may help inform strategies to address key bottlenecks in eel aquaculture, particularly those related to egg quality and larval viability.

## Supplementary information

Below is the link to the electronic supplementary material.

ESM 1 (DOCX 116 KB)

## Data Availability

All primer sequences are included in the supplementary information. All raw data can be made available on request.
