# Supplementary material for: Lipoprotein biology during induced oogenesis in the short-finned eel, Anguilla australis—hepatic target gene expression and accumulation of nutrient sources into developing oocytes
Source: Fish Physiol Biochem. 2026 Oct 3;52(5):184. doi: 10.1007/s10695-026-01806-w (PMC13634022; doi:10.1007/s10695-026-01806-w)
Supplement: Supplementary file 1 [file 10695_2026_1806_MOESM1_ESM.docx]

**Supplementary Fig 1** Relationship between oocyte diameter and eye index (A), hepatic apolipoprotein b protein (B) and relative hepatic mRNA expression of *apolipoprotein e* (*apoe*) and *androgen receptor alpha* (*ara*) in female shortfinned eels (*Anguilla australis*). Eels were treated for 10 weeks with either 10 mg/kg salmon pituitary homogenate (filled circles) or Ringer (open squares), initial controls are indicated by open circles. Sample size: n = 4 (6, 8 and 10 weeks) or n = 5 (all other sampling times)

**Supplementary Table 1** The primers used for PCR amplification of large cDNA fragments of each target gene (*apolipoprotein b* and *e* (*apob* and *apoe*), *androgen receptor alpha* and *beta* (*ara* and *arb*), *elongation one alpha* (*ef1a*), *estrogen receptor one* and *two* (*esr1* and *esr2*), *microsomal* *triacylglyceride transport protein* (*mttp*), *peroxisome proliferator-activated receptor alpha*, *beta* and *gamma* (*ppara*, *pparb* and *ppary*), *ribosomal* *protein L36* (*rpl36*) and *vitellogenin* *Aa* and *Ab* (*vtgAa* and *vtgAb*) from the shortfinned eel, *Anguilla australis*. The length of the amplicons in base pairs, annealing temperature in degrees Celsius (°C) for standard PCR, the accession number or in-house transcriptome contig number, the associated reference and the nucleotide sequence of the individual primers are shown

| Gene name | Accession number | Amplicon size (bp) | Annealing temp (^o^C) | Forward primer | Reverse primer | Reference |
| --- | --- | --- | --- | --- | --- | --- |
| *apob* | Unigene32630 (liver) | 143 | 62 | gatgtgcctcagacatgcag | Gtattctttgccattgcagctt | Damsteegt et al 2014 |
| *apoe* | KJ639930 | 192 | 59 | ggagagagatggacaccctg | Cgttgacgtactgggtggag | Damsteegt et al 2015 |
| *ara* | AB710174 | 882 | 57 | agcggctgcaattcgggacaa | Tccttcccttgagacaacacc | Ozaki et al 2019 |
| *arb* | AB710175 | 840 | 55 | aacagttgctccgagtcagcc | Cgactccgatgcagatctatc | Ozaki et al 2019 |
| *ef1a* | HM367094 | 703 | 55 | aagcagctcattgtgggagt | Aacattgtcaccgggagg | Thomson-Laing 2017 |
| *esr1* | CL8366.contig1 (eye, IR & pit) | 743 | 55 | cgggagaaacgcaaagag | Cctttgttgctcatgtgtcg | Thomson-Laing 2017 |
| *esr2* | Unigene42711 (eye, IR & pit) | 799 | 58 | tccgcaagtgctatgaagtg | Atgtgggccagaagcattag | Thomson-Laing 2017 |
| *mttp* | KJ639932 | 112 | 60 | ttcaccacccacagtcagg | Cagagtttgcctggacacg | Damsteegt et al 2015 |
| *ppara* | HQ259218 (*A. japonica*) | 326 | 53 | cgacaagtgtgaacgcaact | Ccatgtcgtgaatgacgaag | Designed for this study |
| *pparb* | HQ259220 (*A. japonica*) | 433 | 57 | gactgcttgccagagagacc | Aacccgtccttgttcatcag | Designed for this study |
| *ppary* | HQ259217 (*A. japonica*) | 482 | 55 | ctgacctggatcacatgcac | Gatctgcccgtaggagatca | Designed for this study |
| *rpl36* | HM357467 | 289 | 53 | tatggccgttggccttaata | Ggcagcagcctttctcatag | Designed for this study |
| *vtgAa* | AY423445 (*A. japonica*) | 599 | 55 | caccatgttcacaaccttcg | Gcctgaagaactggcagaac | Designed for this study |
| *vtgAb* | AY423444 (*A. japonica*) | 506 | 53 | gctgaagcctaccatcttgc | Acacatgctgtcaacccaaa | Designed for this study |

| Gene name | Accession number | Amplicon size (bp) | Annealing temp (^o^C) | Forward primer | Reverse primer | Efficiency (%) | Reference |  |
| --- | --- | --- | --- | --- | --- | --- | --- | --- |
| *apob* | Unigene32630 (liver) | 143 | 62 | gatgtgcctcagacatgcag | gtattctttgccattgcagctt | 96.1 | Damsteegt et al 2014 |  |
| *apoe* | KJ639930 | 192 | 59 | ggagagagatggacaccctg | cgttgacgtactgggtggag | 96.6 | Damsteegt et al 2015 |  |
| *ara* | HM364283 | 90 | 62 | aggaagaactgcccctcttg | atttgcccgatcttcttcag | 101.5 | Setiawan et al 2012 |  |
| *arb* | HM364284 | 98 | 62 | gcttggagctcgaaaattga | ttggagagatgcactggtg | 96.7 | Setiawan et al 2012 |  |
| *ef1a* | HM367094 | 152 | 62 | cccctgcaggatgtctacaa | agggactcatggtgcatttc | 98.4 | Setiawan and Lokman 2010 | |
| *esr1* | CL8366.contig1 (eye, IR & pit) | 116 | 62 | ggcatggccgagattttc | gcaccggagttgagcagtat | 101.1 | Thomson-Laing 2017 |  |
| *esr2* | Unigene42711 (eye, IR & pit) | 138 | 62 | aaagatccctgggtttgtgg | aggtctggggaaaagatgagtt | 96.3 | Thomson-Laing 2017 |  |
| *mttp* | KJ639932 | 112 | 60 | ttcaccacccacagtcagg | cagagtttgcctggacacg | 97.5 | Damsteegt et al 2015 |  |
| *ppara* | ON844096 | 97 | 60 | tccagaagtgcctgtcagtg | gtgagaatctccgccttcag | 101.5 | Designed for this study |  |
| *pparb* | ON844097 | 147 | 62 | gtctggaaccagctcatcaa | tgaggaagaggtcgacgaag | 98.5 | Designed for this study |  |
| *ppary* | ON844098 | 138 | 62 | ggagctacgcttcttccaca | atgaccccgtacttgagcag | 100.4 | Designed for this study |  |
| *rpl36* | HM357467 | 160 | 60 | cctgaccaagcagaccaagt | tctctttgcacggatgtgag | 96.3 | Setiawan and Lokman 2010 |  |
| *vtgAa* | ON859130 | 130 | 60 | gaagtcactattggtatgac | tttcctcctcatctttcagagtg | 97.4 | Designed for this study |  |
| *vtgAb* | ON859131 | 153 | 60 | cctttgctgtggcaagaaat | catcattataaatgatttcagatgacct | 95.1 | Designed for this study |  |

**Supplementary Table 2** The primers used for quantitative PCR amplification of each target gene (*apolipoprotein b* and *e* (*apob* and *apoe*), *androgen receptor alpha* and *beta* (*ara* and *arb*), *elongation one alpha* (*ef1a*), *estrogen receptor one* and *two* (*esr1* and *esr2*), *microsomal* *triacylglyceride transport protein* (*mttp*), *peroxisome proliferator-activated receptor alpha*, *beta* and *gamma* (*ppara*, *pparb* and *ppary*), *ribosomal* *protein L36* (*rpl36*) and *vitellogenin* *Aa* and *Ab* (*vtgAa* and *vtgAb*) from the shortfinned eel, *Anguilla australis*. The length of the amplicons in base pairs, annealing temperature in degrees Celsius (°C) for standard PCR, the accession number or in-house transcriptome contig number, the associated reference and the nucleotide sequence of the individual primers are shown.

**Supplementary Table 3** The gene and chromosome accession numbers for members of the large lipid transfer protein family (*apolipoprotein b* (*apob*), *microsomal* *triacylglyceride transport protein* (*mttp*), and *vitellogenin* *Aa* and *Ab* (*vtgAa* and *vtgAb*)) from the European (*Anguilla anguilla*) and Japanese eel (*Anguilla japonica*) that were used for analysis of the promoter regions

| Species | Gene | Gene accession number | Chromosome | Chromosome accession number |
| --- | --- | --- | --- | --- |
| *A. Japonica*  *A. anguilla* | *apob* | -  XM_035410564.1 | 1 | CM045883  NC_049201 |
| *A. Japonica*  *A. anguilla* | *mttp* | -  XM_035416929.1 | 5 | CM045887  NC_049205 |
| *A. Japonica*  *A. anguilla* | *vtgAa* | AY423445.1  - | 4 | CM045886  NC_049204 |
| *A. Japonica*  *A. anguilla* | *vtgAb* | AY423444.1  - | 4 | CM045886  NC_049204 |

**Supplementary Fig 2** The locations of estrogen (ERE: GGTCAnnnTGACC highlighted in yellow) and androgen (ARE: GGTACAnnnTGTTCT highlighted in blue) response elements located within the 2kb upstream region of the start codon for *apolipoprotein b* (*apob*), *microsomal triacyglyceride transfer protein* (*mttp*) and the two *vitellogenin* isoforms (*vtgAa* and *vtgAb*) in the European (*A. anguilla*) and Japanese eel (*A. japonica*). Full response element sites are underlined and mismatches are in bold. Exon 1 is highlighted grey and if the TATA-box was identified it was highlighted green.

*Anguilla japonica* apolipoprotein b (*apob*) exon 1, 50 bp of intron 1 and 2kb upstream sequence

ACTGCCACTTTGATCTCGGTCATATATTTCCGCATATAACAATGGTATTTAGTAAACATTTCCATTTATTGACTTGCATTGGAAATATATATTTATATAAAATGTATTGCAATATATATATATATATATATTTTTTCTTTTTTTTTCCTGCACAGGGAAATGACCTGGTGGCTATAGGGCTTAAGGGCAGGAGCTTTGGTTGGTCATTAATAATTAACATCAGAAAAGGACGAAGTACAGTAGCAGCTAAAATGTGGGTGTCTTTTTTGTTTTTTTATCAGTGCCATTTCTCACGATGAGGCTCACATTCTGGCCAAAACACTCAAAACTCCCAGAGTTTAAAAATAAATCCCCAATCCTTTGTAAGCAACACCTATCCACAAAGCATTAATTTGGTCATAAGTTACGTCATTACCTTTCCGTGCTCAGTGTACGCGTGCACAATCCATAGGGCAGGCAAAGATACAGAGTCGTTGTTTGTTTTCTTTTTTTCTTCTTTTTTTTTATCTGAGTAGAACCTTTTAAAACTCCTGGAACATGACACACTGGCCCGGATTAATAGAGTGTTTTAAGGACAGCTTGCTGTGCGGATGGATTTTATGGCATTTTATCTGGAAATCTGTAAAAGAAAAATGTCACTTTTAGACCTCACCCTCCACACCTTCCTCTCCCTATTCATAATATTTTTGATGTTTCCTTTTTCCTGGATTATCCTAAAGGAATATGGAATATTTTGCACTATTAATAAAAAAGGGCATTCATTTATATTTATGTGTGTGTGTGTGTGTGTGTGTGTGTGTGTGTGTGTGCATGCATGTGTTTCTCTAAGGAAGGTGTGACATAACTTTGGCTCTTCAGTATGACTAACGGTTTTACAACACCTTTGCCTGTGACTCTCCTCTGCATCCACCACACCATCCAAAAAGAAAGGGCACTAAAATCTCACTGCGTTTGTCATGTAGCACTTTTGTTTTTTTTAAAAACACTTTAATTGAACGCTTTCTTTTTCTATTTTCTTAATTAGGTAGATGGAAATGCATTTATTCTGATAATATATTTCCAAGTATCAATGAAGACTTGTTGTTTGTGTGCTGTGCATCACCAGTGGATTTTTCTGTATATGAAAAAGAAAATTTTCATAATAGTGAAGTTTATAACAGTTGAGAAAGCAATGAGAAATATTTTAGGGAAAAAATCCACTATGATGTTTACAAACCCTTTGAAGAGTAGTTTTTTTAAAAAAATGTCTTTTTCATAATTTAAAATCAGTATTCTCAAACTCCATTGCTTTATTTACCTGAAGTAATTGTTATATCAGCGTTAGAAGGTTCAATTACTAAGACCATTCTAATTTCGTATTTACAGCCTTGCACCTTAAAGGGTTAAATTAATATTTTGAGCTTTTGAAGTGAGATTGTAGAAGACTGTGGACTATATTGAATAAAAAACAAAACGTGGGTTAAAAAACATGCAACATATAAAGCATATGATTCCATTGAACCTGGAGTCCAGCATATGGACCTCATATGCTATGCTCCTAAAACATGACTCCCTTTACAGCTGTGAAATGGATTTAATGTCCTCAACAGCTTGCTCTGATGTTCAAATGATACCTGTACTGCCTCAGTCTGAACCAGACTCAGTCTTTTTTGCAAGGGGAAAAAAAAAGAGTCAAAATCAAACATAAAAATCTGATTCCAGTATTAACTGTCCCGTAGCCGAAGAGCTCCAGCTTATTGCGTCCTGTTTTTCTGAAACCGGTCGCCTTTGTCCCACCAGAAGGGCTGAACTTTGGAATGCCCTGCTGCAAATGTCTTTTTTTTTTTTGTAGCTGCAACTCTCGATTGATCTCCCTTTGTCCCATCACTGTCACACGGGTGGGGCTGGGCCCGGGCCCCGGGGTATAAAAAGACAGCGCCGGGGCGAGCACACAATCAGTTTGGGTCGGAAAGGAGAGCTCCCTGAGCCCAATTAAGGAACCGAGCTTTCCAGAACCCGTCATGGGTCGATCAAAGCTCTGCCTACTGCTGCTCCTGAGCACCTGCGCCTTGGCCCGTAAGTACTCCGGTGTCCCGAGCGAGAGAGAGAGAGTCCGGTCCTGCCTA

*Anguilla anguilla* apolipoprotein b (*apob*) exon 1, 50 bp of intron 1 and 2kb upstream sequence

ACTGCAAAATATATCAAATACTGCCACTTTGATCTCAGTCATATATATTTCCGCATATAACAATGGTATTTTGTAAACATTTCCATTTATGATATATATTTATATAAAATGTATTGCAGTATATATATATATAATTCTTTTTTTTTTTTTTTTTTTTCCTGCACAGGGAACTGACCTGGTGGCTATAGGGCTTAAGGGCAGGAGCTTTGGTTGGTCATTAATAATTAACACCAGAAAAGGACGAAGTACAGTAGCAGCTAAAATGTGGGTGTCTTTTTTTAAATTTTTTTTAATTTGTGCCATTTCTCACGATGAGGCTCACATTCTGGCCAAAACACTCAAAACTCCCAGAGTTTAAAAATAAATCCCCAATCCTTTGTAAGCAACACCTAGCCACAGAGCATTAATTCGGTCATAAGTTACGTCATTACCCTTCAGTGTACGCGTGCACAATCCATAGGGCAGGCAAAGATACAGAGTCGTTTGTTTTCTTTTTTTCTTCTTTTTTTTTTTTATCTGAGTAGAACCTTTTAAAACTCCTGGAACATGACACACTGGCCCGGATTAATAGAGTGTTTTAAGGACAGCTTGCTGCGTGGATGGAATTTATGGCATTTTATCTGGAAATCTGTAAAAGAAAAATGTCACTTTTAGACCTCACCCTCCACACCTTCCTCTCCCTATTCATGATATTTTTGATGTTTCTTTTTTCCTGTATTATCCTAAAGGAATATGGAATATTTTGCACTATTAATAAAAAAAGGGCATTCATTTATATTTAAGTGTGTGTGTGTGTGTGTGTGTGTGTGCGTGCATGTGTTTCTCTAAGGAAGGTGTGACATAACTTTGGCTCTTCAGTGTGACTAACGGTTTTACAACACCTTTGCCTGTGACTCTCCTCTTCGTACACCAAACCATCCAAAAAGAAAGGGAACTAAAATCTCACTGCGTTTGTCACATAGCACTTTTGTTTTTTTTAAAACACTTTAATTGAACGCTTTCTTTTTCTATTTTCTTAATTAGGGAGATGGAAATGCATTTATTCTGATAATATATTTCCAAGTATCAATGAAGACTTGTTGTTTGTGTGCTGTGCATCACCAGTGGATTTTTCAGTACTGACAAGAAAATTTTCATAATAGTGAAGTTTATAACAGTTGAGAGGGCAATGAGAAATATTTTAGGGAAAAAATCCACTATGATGTTTAAAACCCTTTGAAGAGTAGTTTTTTAAAAATGTCTTTTTCATAATTTAATCAGTATTCTCAAACTCCATTGCTTTATTTACCTGAAGTAATTGTTATATCAGCGTTAGAAGGTTCAATTACTAAGACCATTCTAATTTTGTATTTACAGCCTTGCACCTTAAAGGGTTCAATTAATATTTTGAGCTTTGACTGAAGATTGTAGAAGACTGTAGACTATATTGAATAAAAAACAAAACGTGGGTAAAAAAAAAACATGCAACATATAAAGCATATGATTCCATTGAACCTGGAGTCCAGCATATGGACCTGATACGCTATGCTCCTAAAACATGTCTCCCTTTACAGCTGTGAAATGGATTTAATGTCCTCAACAGCTTGCTCTGATGTTCAAATGATACCTGTACCGCCTCAGTGTGAACCACATTTTTTGCGAGGGGAAAAAAAAAAGAGTCAAAATCAAACATATAAAAATCTGATTCCAGTATTAACTGTCCCGTAGCCGAAGAGCTCCAGGTTATTGCGTCCTGTTTTTTAGAAACCGGTCGCCTTTGTCCCACCAGAAGGGCTGAACTTTGGAATGCCCTGCTGCAAATGTCTTTTTTTTTTTTTTTTTTTTTGTAGCTGCAACTCTCGATTGATCTCCCTTTGTCCCGTTACTGTCACACGGGTGGGGCTGGGCCTGGGCCCCGGGGGTATAAAAAGACGGCGCAGGGGCGAGCACGCAATCAGTTTGGGTCGGAAAAGGAGAGCTCCCTGAGCCCAATTAAGGAACCGAGCTGTCCAGAACCCGTCATGGGTCGATCGAAGCTCTGCCTGCTGCTGCTCCTGAGCACCTGCGCCTTGGCCCGTAAGTACTCCGGTGTCCCGAGCGAGAGAGAGAGAGAGAGAGAGAGAGAG

*Anguilla japonica* microsomal triglyceride transport protein (*mttp*) exon 1, 50 bp of intron 1 and 2kb upstream sequence

TTAATGCCCTGTAATATAATCAAGTTGGAGAGAGCGGCTTCTGGCTCCAATTACAAGTAATAGAGTCCCTGAGCAAACAATGGGCACCGAGAGCAGTGGGATTACATTAAACTGAGGTGCTCAGCAGACTTGGGAGAATTACAAAACACTGAGAGTCCAGTGCTGGCTTGAGCCAAGATGGAGTGTAAACAGGCGGGTTTCCAGTCCGTGGTGTGTGTATGCTGTGTACAGTCTGGCCTTCAGCCCTGGCCCCGGTTTCCACACCGTCCGATCTTACAGCGCTGAGTCACCGCCCCTGCCGCGGGT**G**ACCTTTGACCTTTGACCCTATAGCCCGAGCACCGGCACCAGACGGAGAGAGAGAGAGAGAGCGAGCTTGGCACGTTTTTTCCAGAAGGAGAACGAGCATACCCTGGCGAGCGACCTGGCCCTGTTTTGGTATCCATGGCAACGGCGGCCAGGTGGGGAACCTGTCAGGGTCAGCT**G**GAC**T**CCTGTAAGAGTTCCGGAACCAAAACAATCAACCCGGGCTGTTAGACTCCTCACAGCCTCTTACTGGGGCCCACATTACTTTTTAAAATTATGCATATGGGCCTGATATTTTCCACATACTATTTAGAAAGCAACGAATGTATAAAATTTACTGTACTGTAGTAAATTAATGTAGGTGCCCTTTAAATATGATTAGCCAAGAGTTCTTTTTCTGCCTCTAATGGGGGGATACAGACACTGTGCGAATACAAACCTAATCATTCCATTTCATTAAGACAGTTTCAATAATTAGAATTTTATAGTTTTGCAATGAAGTGGTCTGAAGTGGTAGTTTGTTCTATTAAAATATACACTATTATCATAATCTATAAAAGTATAAATAGGTTAAAAAGCTTTTTCAATATTCTGTGGAAGGTCAAAACAAATGTTGGCCGGCATTGTTCATGTATTTAACTTTTGTTTTGACCCTCCATCTGTAAACACACACATTACGTTGCAGCTACATCAAGGAGATACCCTATACTAATTTTTGACTGAACGTTAAACGTCAAAATAAAATAAAAAATATCGTCGATTTTCACTTATCTCGTTCTTCTTTTGCTTTACAAAAACTGTTTCTATCAGAAGGCAAATAATTATCTGCTAAAGGTGGAG**C**GTAC**C**GATTGTTCTGTATCCCGGAATCTGCATATCGAACTCTGCCAAGGATTTTTTTTTTTATCCCGCGAGGGATGCGTCAGGGAACGTAACGTCAGCGGAGTAGTTTGACCCTGCGGCACCCGCACACGGCGATTCCGCAGCGCTCAACCGCGGAAAATTAGGTTAAAGGTTTACGAACTGATACTATAGCGATACTAACAGAGGAACAGTCGGAACTGAGATGCCAATCCGTTTTCTTTTGTCAGGGAGAATGACCTTTGTAGCAGCTGTTGCTTCCCAGTCTAGCTGAGGAATTTAAGAAGTTACCGAACCGCGCCATACGGTTTTAATCATAGGTTGCTAGTAAGCTAGTTGTTTCCGGATTTGTGCATTTTGTGCTAATCCTCACATCTTCATTTGCATTCCCTGTTTACAGTTCTGATCAAATGAACATACTTTAAATGCGATTGAATGCGAATCTGTTACAACACAGTGCAAAACAGGGGGCGGTGGAAGATTAAATATAATTATTTGACGCTAAGTACCGTCGTCTTGGCTATGCTTGATAAAACCAGATCAATCAGCGATAAGTGGGGGGGACTGTCGCATCCGCAAGACGCTATACTTTATCTTGGGCATGGACTCCGCGGGAACTGCGCCTATCAGAAAGCGAAGCGGTTCTCAAATCTCAGCTCCCCGGGTTAATGATTGAAAGCGTGAACTTAGTACTTGAGCGTGGAGTTTGGAGTCTGGCCTCCACCACGATAAACATTAGCGGAGTATTCCCGAGCGCCCGTCCTCTTTCTCGCGACCCGAGGTGCTGCTCAGTCACTGACTTCTGCTGGTGATTTGCTGTCTGACCGCACAGAGAGCGCACTATGTTAAAGCTAAGCTTAATTTTACTCTGCGCTCTTGCATCGTCCGTGTCTGTCCCCTCGCTAAAGGTGAGTGGTTTACACAATTCATTTGGCTGTAAAATGATTATGAATGGTTTT

*Anguilla anguilla* microsomal triglyceride transport protein (*mttp*) exon 1, 50 bp of intron 1 and 2kb upstream sequence

AAGGCGGAGAGCGGCGTCTGGCTCCAATTACAAAGTAATAGAGTCACTGAGCAAACAATGGGCACCGAGAGAAGTGGGATTACGTGAAACTGAGGTGCTCAGCAGACACGCTCATCCAGGGAGAATTACAAAACACTTAGAGTCCAGTGCTGGTTTGAGCCAAGATGGAGTGTAAACAGGCGGGTTTCCAGTCCGTGGTGTATGTATGCTGTGTACAGTCTGGCCTTCAGCCCTGGCCCCGGTTTCCACCGTCTGATCTTACAGGAGGAGAGCGCTGAGTCACCGCCCCTGCCACGGGTCACCTTTGACCTTTGACCCTATAGCCCCGAGCACCGGCACCAGACGGAGAGAGAGAGAGAGAGAGAGAGGGAGAGAGAGCTCGGCACGTTTTTTCCGGGAGGAGAACGAGCATACCCTGGCGAGCGACCTGGCCCTGTTTTGGTATCCATGGCAACGGCGGCCAGGTGGGAAACCTGTCAGAGTCAGCTGGACTCCTGTAAGAGTTCCGGAACAATAGACCCGGGCTGTCAGACTCCTCACAGCCTCTTACTGGGGCCACATTACTTTTTAAAATTATGCATATGGGCCTGATATTTTCCACATATTATTTAGAAAGCAACAAATGTATAACATTTACTGTACTGTAGTAAATTAATGCAGGTGCCCTTTAGCCAAGAGTTCTTTTTCTGCCTCTAATGGGAGTGATACAGACACTGTGCGAATACAAACCTAATCATTCCATTTCATTAAGAAAGTTTCAATAATAAAAATGTATAGTTTTGCAATGAAGTGGTCATTTGTTCTATTAAAATGAACACTATTATCATAATCTATAAAAGTATAAACAGGTTAAAAAGCTTTTTCAATATTCTGTGGAGGGTGAAAACAAATGTTGGCCGACATTGTTCATGTATTTAACTTTTGTTTTGACCCTCCGTCTGTAAACACACACATTACGTTGCAGTACATCTAGGGGATACCCTATACTAATTTTTGACTGAACGTTAAACGTCAAAATAAAATAAAAATATCGTCGATTTTCACTTATCTCGTTCTTCTTTTGCTTTACAAAAAACTGCTTCTTTTGCTTTACAAAAAACTGTTTCTTATCAGAAGGCAAATAATTATCTGCTAAAGGTGGAG**C**GTAC**C**GATTGTTCTGTATCCCGGAATCTGCATATCGAACTCTGCCAAGGATTTTTTTTTATCCCGCGAGGGATGCGTCAGGGAACGTAACGTCAGCGGAGTAGTTTGACCCTGCAGGACCCGCACACGGCGATTCCGCAGCGCTCACCCGCGGAAAATTAGGTTAAAGGTTTTCAAACTGATACTACAGCGATACTAACAGAGGAACAGTCGGAACTGAGATGCCAATCCGTTTTCTTTTGTCAGGGAGAATGACCTTTGTAGCAGATGTTGCTTCCCAGTGTAGCTGAGGAATTTAAGAAGTTACCGAACCGCGCCATACGTTTTTAATCATAGGTTGCTAGTAAGCTAGTTGTTTCCGGATTTGTGCATTTTTTGCTAATCCTCACATCTTCATTTGCATTGCCTGCTTACAGTTCTGATCAAATTAACATACTTTAAATGCGATTGAATGCGAATCTGTTACAACACAGTGCAAAACAGGGGGCGGTGGAAGATTAAATATAATTATTTGACGCTAAGTACCGTCGTCTTGGCTATGCTTGATAATACCAGATCAATCAGCGATAAGTGGGGGGGCTGTCGCATCCGCAAGACGCTATACTTTATCTTGGGCATGGACTCCGCGGGAACTGCGCCTATCAGAAAGCGAAGCGGTTCTCAAATCTCAGCTCTCCGGGTTAATGATTGAAAGCGTGAACTTAGTACTTGAGCGTGGAGTTTGGAGTCTGGCCTCCACCACGATAAACATTAGCGGAGTATTCCCGAGCGCCCGTCCTCTTTTTCAGGACCCGAGGTGCTGCTCAGTCACTGACTTCTGCTGCTAATTTGCTGTCTGACCGCACAGAGAGCGCACTATGTTAAAGCTAAGCTTAATTTTACTCTGCGCTCTTTCATCGTCTGTGTCTGTCCCCGCTAAAGGTGAGTGATTTACACAATTCATTTGGCTGTCAAATGATTATGAATGGCTTT

*Anguilla japonica* vitellogenin Aa (*vtgAa*) exon 1, 50 bp of intron 1 and 2kb upstream sequence

AATCATAACATAATCATATATTCCACTAAAAATCAACTGTTTTGAGACATGAAACTTACTTTTGCATCATTTTGAACTGGGGATTATTCTCTTCATATAGGGGTCTAAGTTATCTAGGGGTCCAAATACCTATCCAAAACCAAAATAGATACAGTATATGGCCAAAAGTATGTGGACACCCCTTCAGTTAGTGGGTTTGGCCACTTTAGCCACACCAGTTGCTAACTGGTACATAAAATCAAGTGTACAACCATGCAATCTCCATAGACAAACATTGGCAGCGGAATTGCAGCTGAAGAGCTCAGTGACTTTCAGCAGCGGTCATTATG**T**CCCAATAGCTGACCACTTCATCGGGGATTATCTTTCACATATTGTATGACAGGGTGGTGATGCTTAACCACCCCTGTAGTACATTATTTTTCAATGACAGGACAGCACAGAGCAGTTTATTTCACTTATATTAGTTACTTTTATCATTATTGATTTTTATCAACTTAATCAGCTGAAATTGAAATATTCTTCCATAAATTTGATTAATTTGTTGACTTATTTTCTAAGGGGGCAACTGGTTGATTGAGTAACTCAGGGGGTAATTATTTTTTCACACGGGTGATATGAGTGGTTGATAACTTTTTTCATTAAATAAAATGAACTAATAATTTAAAAATGTGTTTTGTGTTTACTCACTTTCCCTTGGTCTGATATTGGATTGTGTCTAAACAATTCAGTGTGACAAATATGCAGTAATAAAGGAAATCAGGAAAGGGGCCTGTTTGAATATGAAATAAATTAAACTGAGTGTGGTCTACCTGGTATTTTTTAGCAAAATAAAATGAAGAAATAAAAAGCACATTTGTAAATACATACATAATATACATGCACACTGTTATAGATAATTTGGCATTGGAGGATGTCACTAGAAATAAATAAGCAAGCACTATGTATTATTTATATCATATATGACCATTAATATTTCAAACTTTTATTCCATATTTATTTATTTTAATGCCACCCTGGGCTCCTTAAATTATGAAAACAGATTGACCAAATTTAATGAAATCCACAAAAAATACTATTATTACTACTACTACATTAATACTATTATTGTGAGCAGTGACACATCAGCCAAACTAGCCAGTTTAATTGGGTGCTCCCAGTAATACAAACACATACATTCAAAGAATTTAATTATTAGATCTTAATTCATTTGTATACACTTCTTCATGCATTACCAACCAGTGCAACCAGTATTAGAGCAATGTAACAATATAAGTAAAATAAAAAATAGTTAATTCATACATTACAATTTCACCTTTCAAAAACATACTCTGGTGGCAAGTAAAAATAACTGACAAACATTAATACGTTCAGAAAATATGACTTAAAATAAAGGTAGAAAAATATTTTTGCATCAGGTAAGCTACATGAACTTGAAATTATTTGGTCCTCCAATTATTAATGTTAATACTGTGCTTCCATCTTGCCTGACATGTATACAATGAATAGTTACAATGAATACAAATGAACAAGACCTAGAAAAAATATATATTATTCCAGTTTTAATTTCACTGTCAATTCTTGTAGTCATAACAATGAGAAATTAGTTATTGAACATTTTTATATTTTATGTTTTTGTTGCCTAAAAGAGTTCAAACAAATTTCTAAAATAGCACAGCAATGCTAATTACAATTCACAAATGGTGACAAAATCTTTCAACTTGGACATTGAACTTGACATCTTATGGAAGCCAACACAATAGGTCAGGTT**AC**CCTAGCCTGAAATAGATTTGACCAACTTCAGCATGTTGTGGTGTCCAATTTGTTTGTAAAACTTCTGTTGTGCTAGATAAACAGCAAACAAAATTTCTCCAACCAGTTTGCAATCTGACAGTGATTTAGACTCAGATTAGTTAATGATATGTTTCTGACAGGTGTGCAGTCAGCAAAAAATTTATTTAAAAAGGCAGTGTATGTCCAGGAGTCTTCACATCCCTTTGGCCATGAGAGCAGTTGTTCTTGCCCTGACTCTAGCCCTTGTGGGTAAGTACATGGACATTTTCAGTTCTGGTCTTGTGAAATTTGAAAATAAT

*Anguilla anguilla* vitellogenin Aa (*vtgAa*) exon 1, 50 bp of intron 1 and 2kb upstream sequence

ATTTTGAACTGGGGATTATTCTCTTCATAAGAGTCTAAGTTATCTAGGGGTCCAAATACCTATCCAAAACCAAAATAGATACAGTATATGGCCAAAAGTATGTGGACACCCCTTCACCCCTTAGTGGGTTTGGCCACTTTAGCCACACCACTTGCTAACTGGTACATAAAATCAAGTGTACAACCATGCAATCTCCATAGACAAACATTGGCAGTGGAATTGCAGCTGAAGAGCTCAGTGACTTTCAGCAGCGGTCATTATG**T**CCCAATAGCTGACCACCTCATCAGGGATTATCTTTCACATATTGTATGACAGGGTGGTGATGCTTAACCACCCCTGTAGTACATTATTTTTCAATGACAGGACAGCACAGTGCGGTTTATTTCACTTATATTAGTTACTTTTATCATTATTGATTTTTATCAACTTAATCAGCTGAAATTGAAATATTCTTCCATAAATTTGATTAATTTGTTGACTTATTTTCTAAGGGGGAAACTGGTTGATTGAGTAACTCAGGGGGTAATTATTTTTTCACACAGGTGATATGAGTGGTTGATAACTTTTTTCATTAAATAAAATGAAATAATAATTTAAAAATGTGTTTTGTGTTTACTCACTTTCCCTTGGTCTGATATTGGATTGTGTCTAAACAATTCAGTGTGACAAATATGCAGTAATAAAGGAAATCAGGAAAGGGGCCTGTTTGAATATGAAATAAATTAAACTGAGTGTGGTCTACCTGGTATTTTTCAGCAAAATAAAATGAAGAAATAAAAAGCACATTTGTAAATACATACATAATATACATGCACACTGTTATAGATAATTTGGCATTGGAGGATTTCACTAGAAATAAATAAGCAAGCACTATGTATTATTTATATCATATATGACTATTAATATTTCAAACTTTTATTCCATATTTACTTATTTTAATGCCACCCTGGGCTCCTTAAATTATGAAAACCAAATTTATTTAAACCAAATTATTGACCAAATTTAATGAAATCCACAAAAAATACTATTATTACTACTACTACATTAATACTATTATTGTGAGCAGTGACACATCAGCCAAACTAGCCAGTTTAATTGGATGCTCCCAATAATACAAACAGACACTCAAGAATTTGAATATGAGAATAAAAAAACAAAATACATTCAAAGAATTTAATTATTAGATCTTAATTCATTTGTATACACTTTTTCATGCATTACCAACCAGTGCAACCAGTATTAGAGCAATGTAACCATATAAGTAAAATAAAAAATAAATAGCAGTTAATTCATACATTACATTTTCACCTTTCAAAAACATACTCTGGTGGCAAGTAAAAATAACTAACATTAATATGTTCAGAAAATATAACTTAAAATAAAGGTAGAAAAATATTTTTGCATCAGGTAAGCTACGTGAACTTGAAATTATTTGGTCCTCCAATTATTAATGTTAATACTGTGCTTCCATCTTGCCTGACATGTATACAATGAATAGTTACAATGAATACAAATGAACAAGACCTAGAAAAAATATATATTATTCCAGTTTTAATTTCACTGTCAATTCTTGTAGTCATAACAATGAGAAATTAGTTATTGAACATTTTTATATTTTATGTTTTTGTTGCTTAAATGCTTCAAAGAGTTCAAACAAATTTTTAAAATAGCACAGCAATGCTAATTACAATTCACAAATGGTGACAAAATCTTTCAACTTGGACATTGAACTTGACATCTTATGGAAGCCAACACAATAGGTCAGGTT**AC**CCTAGCCTGAAATATATTTGACCAACTTCAATATGTTGTGTTGTCCAATTTGTTTGTAAAACTTCTGTTGTGCTAGATAAACAGCAAACAAAATTTCTCCAACCAGTTTGCAATCTGACAGTGATTTAGACTCAGATTAGTTAATGATATGTTTCTGACAGGTGTGCAGTCAGCAAAAAATTTCTTTAAAAAGGCAGTGTATGTCCAGGAGTCTTCACATCCCTTTGGCCATGAGAGCAGTTGTTCTTGCCCTGACTCTAGCCCTTGTGGGTAAGTACATGGACATTTTCAGTTCTGGTCTTGTGAAATTTGAAAATAAT

*Anguilla japonica* vitellogenin Ab (*vtgAb*) exon 1, 50 bp of intron 1 and 2kb upstream sequence

ACTCAGGCAACCTGGTTTCTTGGGTCTGTCACTGATCTTAAGGAAAAATAAAAAACCAACAGGCTCTGTGGCTGTCCAGAAACGGGATTGTGTGTGGTTGCAGGAAAAAGAAAAATGCAACCATATAAAGCAGGGGTGTCCAATCTTATTCAAAAAGTGGTGTGGGTGCAAGTCTTTATTTTAGCCCAGCACTAAGTCATCTGATTCTACTTGATGTTTTGATTTGGAAATGGGAATGGGTTACCTACATAAACTAAAATTACCTACATGAACTACAATCAAGTACACATTTCAATGAATTGAAATATTCCTTTATAATACTTTTTAAGACAGTACTCCTCTTATTGAATTTGAGTTCTCATTTCTTGTATATTTTGCTGTTGTGTGCATTTAGGAAGACTATTTGTATTTGTTTATATTGATAAATCTAGTTTTACAATTTTTCTTAATTTTCATGGGACCCTGTCTATAACAAGATTCATAATTTCAAGAGGCTTTATTTTGTCAAAAATGTTTATATAAAATACCCTTTACACACGCAGAGTAATGTCTTTTGAGAAATGTTTACCCAAATATATTTGTTGCTTTATCACAAATTATGATAAATACTTCAATTCAAAAGCATTCTCTTGAGTTATCCTAAACTTGTGCTTCTGAAGGCCATTGTAAATAAAAAGCAATTGATTGACATCTATCTTGTTCCCTTTGACAAAAATGGTCATTTTGACCTGATATAGCATGTCATGCCAACTGTATTGATTTGTCCAAACAAGTTTGAAGTCATTCTGGCATTCTAAAACAGAAATGATGACTGATCAAAATGTATTCATTTTCTGTTAGTAAAAGGCAGCATGAATGGAAATATAATTATTATGAGAAAATGCTAATTGCAAAACAAAGCTTATTCGATATTTGTGTACCGCGCCCTCCAGAATTATTTATGCCGTTGATAAAGATAATATTACATGGTCACCAAATTGGTAAATTACACTAATACAATGGCTGAGAAAAAGATTTTGTTTAAGATGCTATTTTTCTCCAGAATGATTATTCACACCTTACTAATTATTTTAAATAAAATCCAACTAAAATAATCTGGAATAACATCTTACCATCTTAAAAAAAGAAAAAAAAGATTTTAGGGATCTGTATTGTGGCCTAATGGCATCATGTGGTGCAAAAATAAGTAATCACAATAAAATGCATCATGCATCAATATGGGAAAGGAAATAGTTCTTTAGAAGAAGACAAAGGGTTGTTGACATTTACAAGTCAGAAGATTGTTACAAATAAATAGATACAAAATAAATACCACTTTCAAATTTTAGGTCAATAACTAAGAAGTTTAAAACCACTGGTGTAGTGGTAAAACTGCCTGGTTGAGGACCTGGTATCTATTTTGATTGATACAACTAAATCGTTATTAAAGTTACATATTTTCCCTATTTCAGAATTAAAATATAGTTCATTACTTGCATTATTCCTGTACTAGTCTTTTTGTTCATCTATACCAAACTCTGAATAATTTTGAAGGGTCTGTATCTTTGTATTCTTACTGAATTTAAACAAATACCATAAAATGCATATAAATTAACTTTAAAAAAAATTAACTTTTCATTAATCAAAAGTAAAGCCATGCACTGAACACCATTGAGTACAATTAAACACATAAGATGACATCATATGTCTTGGAATCCATTTAATCTATGAAATCATTTGAAATTATGAAAGTGATTGTACTTTATATAAATTCAGCTCATGAGGAAGGTAAACACAAAAGCCAAGGT**T**AACCT**A**ACCTGTGGTCAACCT**C**AC**T**ATCAATCATTTGTCTATTTATTCAGAACTTTGTGACAGTTAACAGATAACCTTTCTCACTATGTGAAATCTTACAAAGTTATGTGAACTCGAGACGTTAAGTTAATATTACATCTTGTAATACCCTTCGCCAAAATATTTCTATAAAAAGGCAGGTTGTGTCCAGTAGTCTTCACATCCCACCAGCCATGAGAGCAGTCGTCCTTGCCCTGACTCTAGCCCTTGTGGGTAAGTAAAATGCCTTTTATCCCATCTCTGATTTTTCATGTTTGCAGTAC

*Anguilla anguilla* vitellogenin Ab (*vtgAb*) exon 1, 50 bp of intron 1 and 2kb upstream sequence

TCCTTGGGTCTGTCACTGATCTTAAGGAAAAATAAAAAACCAACAGGCTCTGTGGCTATCCAGAAACGGGATTGTGTGTGGTTGCAGGAAAAAGAAAAATGCAACCATATAAAGCAGGGGTGTCCAATCTTATTCAAAAAGTGGTGTGTGTGCAAGTCTTTATTTTAGTCCAGCACTAAGGCATCTGATTCTACTTGATGTTTTGATTTGGAAATGGGAATGGGTTACCGACATAAACTAAAATTACCTACATGAACTACAATCAAGTACACATTTCAATGAATTGAAATATTCCTTCATAATACTTTTTAAGACAGTACTCCTCTTATTGAATTTGAGTTCTCATTTCTTGTATATTTTGCTGTTGTGTGCATTTAGGAAGACAATTTATATTTGTTTATATTGATAAATCTAGTTTTACAATTTTTCTTAATTTTCATGGGACCCTGTCTATGACAAGATTCACAATTTCAAGAGGCTTTATTCTGTCAAAAATGTTTAAATAAAATACCCTTTACACACGCAGAGTAATGTCTTTTGAGAAATTTTTGCCCAAATATATTTGTTGCTTTATCAAAAAGTATGATAAATACTTCAATTCAAAAGCATTCTCTTGAGTTATCCTAAACTTGTGCTTCTGAAGGCCATAGTAAATAAAAAGCAATTGATTGACATCTATCTTGTTCCCTTTGACAAAAATGGTC**G**TTTTGACCTGATATAGCATGTCATGCCAACTGTATTGATTTGTCCAAACAAGTTTGAAGTTATTCTGGCATTCTAAAACAGAAATGATGACTGATCAAAATGTATTCATTTTCTGTTAGTAAAAGGCAGAATGAATGGAAATATAATTATTATAAGAAAATGCTAATTGCAAAACAAAGCTTATTCGATATTTGTGTACCGCTCCCTCCAGAATTATTTATGCCGCTGATAAAGATAAGTAACATTACGTGGTCACCAAATTGGTAAATTATACTAATACAATGGCTGAGAAAAAGATTTTGTTTAAGATCCTATTTTTCCCCAGAACGATTATTCACACCGTATTAATTATTTTAAATAAAATCCCACTAAAATAAATCTGGATTAACATTTTACCATCTTAAAAAAAGAAAAAAATTTTTTTAGGGATCTGTATTGTGGCCGAATGGCATCATGTGGTGCAAAAATAAGTAATCACAATAAAATGCATCTTGCATCAATATGGGAAAGGAAATAGTTCTTTAGAAGAAGACAAAGGGTTGTTGACATTTACAAGTCAGAAGATTGTTACAAATAAATAGATACAAAATAAATACCACTTTCAAATTTTAGGTCAATAACTAAGAAGTTTAAAACCACTGGTGTAGTGGTAAAACTGCCTGGTTGAGGACCTGGTATCTATTTTGATTGATACAACTAAATGGTTACTAAAGCTACATATTTTCCCTATTTCGGAATTAAAATATAGTTAATTACTTGCATTATTCATGTACTAGACTTTTTGTTCATCTATACCAAACTCTGTGAATCATTTTGAAGGGTCTGTATCTTTGTATTCTTACTGAATTTAAACAAATACCATAAAATGCATATAAACTAACTTTTAAAAAAATTAACTTTTCATTAATTAAAAGTAAAGCCATGCACTGAACACCAGTGAGTACAATTAAACACATAAGATGACATCATATGTCTTGGAATCCATTTAATCTATGAAATCATTTGAAATTATAAAAGTGATTGTACTTCATATAAATTCAGCTCATGAGGAAGGTAAACACAAAAGCCAAGGT**T**AACCT**A**ACCTGTTATGGCTGTGGTCAACCT**C**AC**T**ATCAATCATTTGTCTATTTATTCAGAACTTTGTGACAGTTAACAGATAACCTTTCTCACTATGTGAAATCTTACAAAGTTATGTGAACTTGAGGCGTTAAGTTAATATTATATCTTGTAATACCCTTTGCCAAAATATTTCTATAAAAGGGCAGGTTGTGTCCAGTAGTCTTCACATCCCACCAGCCATGAGAGCAGTCGTCCTTGCCCTGACTCTAGCCCTTGTGGGTAAGTAAAATGCCTTTTATCCCATCTCTGATTTTTCATGTTTGCAGTAC

**Reference list**

Damsteegt EL, Falahatimarvast A, McCormick SPA, Lokman PM. (2015) Triacylglyceride physiology in the short-finned eel, Anguilla australis - changes throughout early oogenesis. Am J Physiol Regul Integr Comp Physiol 308, R935-R944. doi.org/10.1152/ajpregu.00436.2014.

Damsteegt EL, Mizuta H, Ozaki Y, Hiramatsu N, Todo T, Hara A, Ijiri S, Adachi S. (2014) Development and partial characterisation of an antiserum against apolipoprotein B of the short-finned eel, Anguilla australis. J Comp Physiol B 184, 589-599. doi.org/10.1007/s00360-014-0821-4.

Ozaki Y, Damsteegt EL, Setiawan AN, Miura T, Lokman PM. (2019) Expressional regulation of gonadotropin receptor genes and androgen receptor genes in the eel testis. Gen Comp Endocrinol 280, 123-133. doi.org/10.1016/j.ygcen.2019.04.020.

Setiawan AN, Lokman PM. (2010) The use of reference gene selection programs to study the silvering transformation in a freshwater eel Anguilla australis: a cautionary tale. BMC Mol Biol 11, 75. doi.org/10.1186/1471-2199-11-75.

Setiawan AN, Ozaki Y, Shoae A, Kazeto Y, Lokman PM. (2012) Androgen-specific regulation of FSH signalling in the previtellogenic ovary and pituitary of the New Zealand shortfinned eel, Anguilla australis. Gen Comp Endocrinol 176(2), 132-143. doi.org/10.1016/j.ygcen.2011.12.041.

Thomson-Laing G. (2017) Effects of reproductive hormones on ovarian development, vitellogenin physiology and gene expression of hormone receptors in the pituitary and ovary of the shortfinned eel (Anguilla australis). (Thesis, Master of Science). University of Otago. http://hdl.handle.net/10523/7686.
